# Detecting Protein-Protein Interactions with a Novel Matrix-Based Protein Sequence Representation and Support Vector Machines

**DOI:** 10.1155/2015/867516

**Published:** 2015-04-27

**Authors:** Zhu-Hong You, Jianqiang Li, Xin Gao, Zhou He, Lin Zhu, Ying-Ke Lei, Zhiwei Ji

**Affiliations:** ^1^College of Computer Science and Software Engineering, Shenzhen University, Shenzhen, Guangdong 518060, China; ^2^Department of Medical Imaging, Suzhou Institute of Biomedical Engineering and Technology, Suzhou, Jiangsu 215163, China; ^3^College of Information Science and Engineering, Guilin University of Technology, Guilin, Guangxi 541004, China; ^4^School of Electronics and Information Engineering, Tongji University, Shanghai 200092, China

## Abstract

Proteins and their interactions lie at the heart of most underlying biological processes. Consequently, correct detection of protein-protein interactions (PPIs) is of fundamental importance to understand the molecular mechanisms in biological systems. Although the convenience brought by high-throughput experiment in technological advances makes it possible to detect a large amount of PPIs, the data generated through these methods is unreliable and may not be completely inclusive of all possible PPIs. Targeting at this problem, this study develops a novel computational approach to effectively detect the protein interactions. This approach is proposed based on a novel matrix-based representation of protein sequence combined with the algorithm of support vector machine (SVM), which fully considers the sequence order and dipeptide information of the protein primary sequence. When performed on yeast PPIs datasets, the proposed method can reach 90.06% prediction accuracy with 94.37% specificity at the sensitivity of 85.74%, indicating that this predictor is a useful tool to predict PPIs. Achieved results also demonstrate that our approach can be a helpful supplement for the interactions that have been detected experimentally.

## 1. Introduction

Since detection of protein interactions is of fundamental importance to understand the molecular mechanism in biological systems, many researchers have focused on this area in postgenome era [1, 2]. Over the past decades, high-throughput experimental techniques, such as yeast two-hybrid (Y2H) system [3, 4] and mass spectrometry (MS), involving genome-wide detection of PPIs, have been developed to generate large amounts of interaction data. However, these traditional experimental methods are time-consuming and expensive, especially for genome-wide scale. In addition, the high-throughput biological experiment usually suffers from high rates of both false negatives and false positives [5]. Combining the experimental techniques with computational model is a promising direction to better understand the mechanisms of protein interactions at the molecular level and to unravel the global picture of PPIs in the cell [6, 7]. Hence, it is of great practical significance to build low cost protein detection systems and establish the reliable computational methods to facilitate the detection of PPIs.

So far, a variety of computational methods have been developed to effectively and accurately predict protein interactions [2, 8–10]. The computational approaches for in silico prediction can be roughly categorized into genome based approaches, network topology based approaches, literature knowledge based methods, and structure based approaches [11]. In addition, there are also some approaches that integrate interaction information from several different biological data sources [9, 10].

However, the aforementioned approaches cannot be implemented if prior information about the proteins is not available [12]. Recently, the sequence-based approaches which derive information directly from protein amino acids sequence are of particular interest [13, 14]. Prediction of protein interactions from only protein sequence is a much more universal way [15, 16]. The previous works demonstrate that the RNA and protein sequences alone contain sufficient information [17, 18]. The previous researches demonstrated that the information of protein amino acid sequences is sufficient to predict PPIs. Although the sequence-based approaches can yield a high prediction accuracy of 80%~88%, it is necessary to design the novel approaches to further improve the prediction performance compared with the existing methods.

In recent years, many efforts have been made aiming to develop accurate approaches for identifying PPIs based on protein sequence information [19, 20]. Shen et al. built a prediction model by employing the conjoint triad feature extraction and support vector machine. When applied to predicting* human* PPIs, this method yields a high prediction accuracy of about 84% [21]. Because the conjoint triad method did not take the neighboring effect into account and protein interactions usually occur in the discontinuous amino acids segments in the sequence, Guo et al. proposed an approach based on SVM and autocovariance feature representation which extract the interactions information in the discontinuous amino acids segments in the sequence [22]. Their approach reached a prediction accuracy of 86.55%, when applied to predicting* saccharomyces cerevisiae* PPIs. Lately, You et al. developed a novel ensemble learning model to predict* Saccharomyces cerevisiae* PPIs from protein primary sequences directly [23]. In this study, the protein pairs retrieved from the database of interacting proteins (DIP) were encoded into feature vectors by using four kinds of protein sequences information. Focusing on dimension reduction, an effective feature extraction method PCA was then employed to construct the most discriminative new feature set. Finally, multiple extreme learning machines were trained and then aggregated into a consensus classifier by majority voting. The experimental results show that it is a very promising scheme for PPIs prediction.

In this study, we report a novel sequence-based method for the prediction of interacting protein pairs using a matrix-based protein sequence descriptors combined with support vector machine (SVM) algorithm. More specifically, we first represent each protein sequence as a feature matrix, from which a novel matrix-based protein descriptor is extracted to numerically characterize each protein sequence. Then we characterize a protein pair in different feature vectors by coding the vectors of two proteins in this protein pair. Finally, an SVM model is established using these feature vectors of the protein pair as input. To evaluate the prediction performance, the proposed method was applied to* Saccharomyces cerevisiae* and* Helicobacter pylori* PPI datasets. The experiment results show that our method can achieve 90.06% and 85.91% prediction accuracy with 94.37% and 83.33% specificity at the sensitivity of 85.74% and 85.27%, respectively. Achieved results demonstrate that the approach can be a helpful supplement for the interactions that have been detected experimentally.

## 2. Materials and Methodology

**Figure 1:**
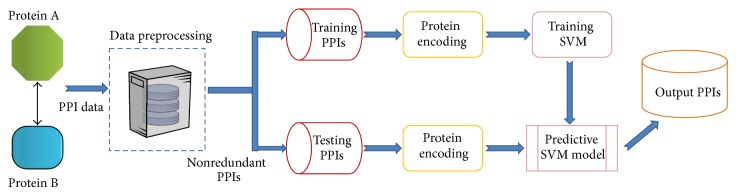
The schematic diagram for detecting protein-protein interactions by integrating experimental PPI data with SVM model.

In this section, we outline the main idea behind the proposed method. The schematic diagram intuitively showing how to detect protein interactions using experimental PPIs data with computational model is given in Figure 1. Firstly, we briefly discuss the PPIs datasets which is employed in the study (the source code and the datasets are freely available at http://sites.google.com/site/zhuhongyou/data-sharing/ for academic use). Next we propose the novel matrix-based protein representation method. Finally, we briefly describe the computational model, SVM, used in this study.

### 2.1. Golden Standard Datasets

We evaluated the proposed method with two real PPIs datasets. The first one was collected from* Saccharomyces cerevisiae* core subset of database of interacting proteins (DIP). After the redundant protein pairs which contain a protein with fewer than 50 residues or have ≥40% sequence identity were deleted, the remaining 5,594 protein pairs comprise the golden standard positive dataset. The selection of golden standard negative dataset has an important impact on the prediction performance, and it can be artificially inflated by a bias towards dominant samples in the positive data. For golden standard negative dataset, we followed the previous work [22] assuming that the proteins in different subcellular compartments do not interact with each other.

After strictly following the steps in Guo's work, we finally obtained 5,594 protein pairs as the golden standard negative dataset. By combining the above two golden standard positive and negative PPI datasets, the final whole PPI dataset consists of 11,188 protein pairs, where nearly half are from the positive dataset and half are from the negative dataset. The second one is a small-scale* Helicobacter pylori* PPIs dataset, which is composed of 2,916 protein pairs (1,458 interacting pairs and 1,458 noninteracting pairs) as described by Martin et al. [24].

### 2.2. Representing Proteins with Descriptors from Primary Protein Sequences

To successfully use the machine learning algorithm to detect PPIs from primary protein amino acids sequences, one of the computational challenges is to effectively characterize a protein sequence by a fixed length feature vector in which the important information content of proteins is fully encoded [25]. In this study, we propose a novel matrix-based protein sequence representation approach for predicting PPIs. Firstly, the protein sequence is transformed into a sparse matrix, which considered the properties of one amino acid and its vicinal amino acids and regarded any two continuous amino acids as a unit. Then the protein features are extracted from the obtained sparse matrix.

A protein sequence can be represented as a series of amino acids by their single character codes A, R, N, D, C, E, Q, G, H, I, L, K, M, F, P, S, T, W, Y, and V. Consider a protein sequence with *L* amino acid residues:

(1) $$\begin{array}{c} S_{1}\;S_{2}\;S_{3}\;S_{4}\;S_{5}\;S_{6}\;S_{7},\ldots ,S_{L}, \end{array}$$

where *S*_1_ denotes the amino acid at protein chain position 1, *S*_2_ denotes the amino acid at protein chain position 2, and so forth. *L* denotes the length of the protein sequence. We scan the protein sequence from left to right by stepping each two vicinal amino acids at a time, which considers the properties of one amino acid and its vicinal amino acid and regards any two continuous amino acids as a unit. Here the number of all possible pairs of amino acids (dipeptides) that can be extracted from the protein sequence is 400, that is, AA, AR, AN,…, YV, and VV.

For step *j*^^ (*j* = 1,2, 3,…, *L* − 1), if the “*S*_*j*_ *S*_*j*+1_” is the *i*th type of dipeptide, then we set the element *a*_*ij*_ = 1. The rest can be done in the same manner and then a protein sequence can be transformed into a 400 by *L* − 1 matrix (see Table 1), namely, *M*, as follows:

(2) Maij400×L−1,aij=1,if  SjSj+1=dipeptidei0,others,

where *L* is the length of protein sequence, *i* = 1,2, 3,…, 400, *j* = 1,2, 3,…, *L* − 1, and dipeptide(*i*) denotes the *i*th type of dipeptides listed in Table 1. Here, each column of the matrix *M* is a unit vector, in which only one element is 1 and the others are all 0. We can see from Table 1 that the occurrence position of all kinds of dipeptides along the protein sequence is contained in the column of the matrix *M*. Meanwhile, the row of the matrix *M* denotes the *i*th kind of dipeptide appearing at the *j*th position within the protein sequence.

Generally speaking, the matrix *M* transformed from protein amino acid sequence embodies the essential information including the information of its sequence order and sequence length of the protein sequence. Thus, given a protein primary sequence, we can design a matrix-based protein descriptor to represent it, which is capable of facilitating PPIs detections.

Low-rank approximation (LRA) is an important matrix analysis method, in which the cost function measures the fit between a given sparse matrix and an approximating matrix (the optimization variable), subject to a constraint that the approximating matrix has reduced rank [26]. Here, using LRA upon the obtained protein feature matrix, we derive a matrix-based descriptor to represent the protein sequence. For a feature matrix *M*, which denotes a 400∗(*L* − 1) matrix, the LRA of the data can be written as follows:

(3) $$\begin{array}{c} \underset{\hat{M}}{\min}\;{\left\|M-\hat{M}\right\|}_{F} \end{array}$$

(4) $$\begin{array}{c} \text{Subject to:}\;\mathrm{rank}\left(\hat{M}\right)\leq r, \end{array}$$

where ‖·‖_*F*_ is the Frobenius norm. The above minimization problem has analytic solution in terms of the singular value decomposition (SVD) of the data matrix *M*.

Let *M* = *U*Σ*V* ^*T*^ ∈ *R* ^*m*×*n*^ be the SVD of *M* and partition *U*, Σ = :diag(*σ*_1_, *σ*_2_, *σ*_3_,…, *σ*_400_), and *N* as follows:

(5) U:U1U2,Σ=:Σ100Σ2,V=:V1V2,

where Σ_1_ is a *r* × *r* matrix, *U*_1_ is *m* × *r*, and *V*_1_ is *n* × *r*. Then the rank-*r* matrix is obtained as follows:

(6) $$\begin{array}{c} {\hat{M}}^{*}=U_{1}\Sigma_{1}V_{1}^{T}, \end{array}$$

where ${\left\|M-{\hat{M}}^{*}\right\|}_{F}=\min_{\mathrm{rank}(\hat{M})\leq r}{\left\|M-\hat{M}\right\|}_{F}=\sqrt{\sigma_{r+1}^{2}+\sigma_{r+2}^{2}+\cdots +\sigma_{m}^{2}}$.

Then we compute the square root of the reduced matrix Σ_1_ to obtain Σ_1_^1/2^ with dimensions *r*-by-*r*. Finally, we can get a 400∗*r* matrix *U*_1_Σ_1_^1/2^, which contains the information of protein sequence order. It should be noticed that the feature matrix *M* for different protein sequences sometime have different columns with each other, which shows that these protein primary sequences are of nonequal length. However, the *U*_1_Σ_1_^1/2^ for different protein sequences are 400∗*r* matrix.

We build a vector (row matrix) from the obtained matrix *U*_1_Σ_1_^1/2^ by concatenating all rows, from 1 to 400, of matrix *U*_1_Σ_1_^1/2^. Therefore, the matrix-based protein descriptor consists of a total of 400∗*r* descriptor values; that is, a 400∗*r* dimensional vector has been built to represent the protein sequence. Considering the trade-off between the overall prediction accuracy and computational complexity for extracting protein sequence descriptors, the optimal rank is *k* = 4. Thus, we set *k* to 4 in this study. A representation of an interaction pair is formed by concatenating the descriptors of two protein sequences in this protein pairs.

### 2.3. Support Vector Machine

Machine learning has been seen as useful and reliable in many applications. Various machine learning techniques can be employed to predict the PPIs. Among them, support vector machine (SVM) is one of the popular learning algorithms based on statistical learning theory [27]. Here we give a brief introduction to the basic idea of SVM.

The goal of the SVM algorithm is to find an optimal hyperplane that separates the training samples by a maximal margin, with all positive samples lying on one side and all negative samples lying on the other side. Suppose that we are given a training dataset of *N* instance-labeled pairs *X* = {(*x*_1_, *y*_1_), (*x*_2_, *y*_2_),…, (*x*_*N*_, *y*_*N*_)} with input data *x*_*i*_ ∈ *R* ^*n*^ and labeled output data *y*_*i*_ ∈ {+1, −1}. The SVM algorithm solves the quadratic optimization problem as minimizing the function as below:

(7) $$\begin{array}{c} \underset{w,b,\xi }{\min}\;\frac{\langle w\cdot w\rangle }{2}+C\overset{N}{\underset{i=1}{\sum }}\xi_{i} \end{array}$$

subject to

(8) yiw·xi+b≥1−ξi,ξi≥0,i=1,2,3,…,N,

where *w* is the normal vector of hyperplane; *b* is the bias of hyperplane; *C* is the penalty factor; *ξ*_*i*_ is the slack variable.

Since ‖*w*‖^2^ is convex, minimizing (7) under linear constraints (8) can be solved with Lagrange multipliers. Further, the aforementioned optimization problem can be transferred to a dual form as maximizing the function

(9) $$\begin{array}{c} L\left(\alpha \right)=\overset{N}{\underset{i=1}{\sum }}\alpha_{i}-\frac{1}{2}\overset{N}{\underset{i,j=1}{\sum }}y_{i}y_{j}\alpha_{i}\alpha_{j}\langle x_{i}\cdot x_{j}\rangle \end{array}$$

subject to

(10) ∑i=1Nyiαi=0,0≤αi≤C,i=1,2,3,…,l,

where *C* ≥ 0, *α*_*i*_ = [*α*_1_, *α*_2_, *α*_3_,…,*α*_*l*_]^*T*^, and *α*_*i*_ ≥ 0, (*i* = 1,2, 3,…, *l*) are coefficients corresponding to *x*_*i*_. *x*_*i*_ with nonzero *α*_*i*_ is called support vector.

In real applications, the training samples are not linearly separable in its original space. Usually, the training samples *x*_*i*_ are mapped into a high-dimensional feature space through some nonlinear function *ϕ*. Then SVM finds a linear separating hyperplane with the maximal margin in this higher-dimensional space. Furthermore, *K*(*x*_*i*_, *x*_*j*_) = *ϕ*(*x*_*i*_)^*T*^ · *ϕ*(*x*_*j*_) is called the kernel function. Actually, the flexibility and classification power of SVM reside in its kernel functions, since they make it possible to discriminate within challenging datasets. Typical kernel functions for SVM include polynomial function, linear function, sigmoid function, and radial basis function (RBF):

 polynomial: *K*(*x*_*i*_, *x*_*j*_) = (*γx*_*i*_^*T*^ *x*_*j*_ + *γ*)^*D*^, *γ* > 0;
 linear: *K*(*x*_*i*_, *x*_*j*_) = *x*_*i*_^*T*^ *x*_*j*_;
 sigmoid: *K*(*x*_*i*_, *x*_*j*_) = tanh(*γx*_*i*_^*T*^ *x*_*j*_ + *B*);
 radial basis function (RBF): *K*(*x*_*i*_, *x*_*j*_) = exp⁡(−*γ*‖*x*_*i*_ − *x*_*j*_‖^2^), *γ* > 0;
 here, *D*, *B*, and *γ* are kernel parameters which are set a priori.

If we replace samples *x*_*i*_ with their mapping in the feature space *ϕ*(*x*_*i*_), (9) becomes

(11) $$\begin{array}{c} L\left(\alpha \right)=\overset{N}{\underset{i=1}{\sum }}\alpha_{i}-\frac{1}{2}\overset{N}{\underset{i,j=1}{\sum }}y_{i}y_{j}\alpha_{i}\alpha_{j}K\left(x_{i},x_{j}\right) \end{array}$$

and the decision function becomes

(12) $$\begin{array}{c} f\left(x\right)=\mathrm{sign}\left(\overset{N_{S}}{\underset{i=1}{\sum }}\alpha_{i}y_{i}K\left(x_{i},x\right)+b\right), \end{array}$$

where *N*_*S*_ is the number of SV, *x* = [*x*_1_, *x*_2_, *x*_3_,…, *x*_*l*_] is the input sample, and *α*_*i*_ and *y*_*i*_ are Lagrange multipliers.

## 3. Results and Discussion

In the section, we describe our simulation methodology and present the experimental results that evaluate the effectiveness of our schemes. The proposed sequence-based PPI predictor was implemented using MATLAB platform. For SVM algorithm, the LIBSVM implementation available from http://www.csie.ntu.edu.tw/~cjlin/libsvm/ was utilized, which was originally developed by Chang and Lin [28]. As the kernels, four kinds of kernel functions, radial basis function (RBF), polynomial function, linear function, and sigmoid function, were selected to implement the experiment. The optimized parameters for the SVM were obtained with a grid search approach. In the simulation, all the experiments were carried out on a computer with 3.1 GHz 2-Core CPU, 12 GB memory, and Windows operating system.

### 3.1. Measures for the Prediction Performance

In the study, fivefold cross-validation technique has been employed to evaluate the performance of the proposed model. In the fivefold cross-validation technique, the whole dataset is randomly divided into five subsets, where each subset consists of nearly equal number of interacting and noninteracting protein pairs. Four subsets are used for training and the remaining set for testing. This process is repeated five times so that each subset is used once for testing. The performance of method is average performance of method on five sets.

Several evaluation measures have been used in the study to measure the predictive ability of the proposed method. The parameters are as follows: (1) the overall prediction accuracy (ACC) is the percentage of correctly identified interacting and noninteracting protein pairs; (2) the sensitivity (SN) is the percentage of correctly identified interacting protein pairs; (3) the specificity (SP) is the percentage of correctly identified noninteracting protein pairs; (4) the positive predictive value (PPV) is the positive prediction value; (5) the negative predictive value (NPV) is the negative prediction value; (6) the *F*-score is a weighted average of the PPV and sensitivity, where an *F*-score reaches its best value at 1 and worst score at 0; (7) the Matthew correlation coefficient (MCC) is more stringent measure of prediction accuracy accounting for both under- and overpredictions. These parameters are defined as follows:

(13) ACC=TP+TNTP+FP+TN+FN,SN=TPTP+FN,SP=TNTN+FP,PPV=TPTP+FP,NPV=TNTN+FN,F1=2×SN×PPVSN+PPV,MCC=TP×TN−FP×FNTP+FN×TN+FP×TP+FP×TN+FN,

where true positive (TP) is the number of true PPIs that are predicted correctly; false negative (FN) is the number of true PPIs that are predicted to be noninteracting pairs; false positive (FP) is the number of true noninteracting pairs that are predicted to be PPIs; and true negative (TN) is the number of true noninteracting pairs that are predicted correctly.

The above-mentioned parameters rely on the selected threshold. The area under the ROC curve (AUC), which is threshold-independent for evaluating the performances, can be easily calculated according to the following formula [29]:

(14) $$\begin{array}{c} \text{AUC}=\frac{S_{0}-n_{0}\left(n_{0}+1\right)/2}{n_{0}\times n_{1}}, \end{array}$$

where *n*_0_ and *n*_1_ denote the number of positive and negative samples, respectively, and *S*_0_ is the sum of the ranks of all positive samples in the list of all samples ranked in increasing order by estimated probabilities belonging to positive. AUC values can give us a good insight into performance comparison of different prediction methods. Although the AUC is threshold-independent, an appropriate threshold must be selected for the final decision. For the classifier which outputs a continuous numeric value to represent the confidence or probability of a sample belonging to the predicted class, adjusting the classification threshold will lead to different confusion matrices which decide different ROC points [21].

### 3.2. Prediction Performance of Proposed Model

We evaluated the performance of the proposed model using the DIP PPIs data as investigated in Guo et al. [22]. To guarantee that the experimental results are valid and can be generalized for making predictions regarding new data, the fivefold cross-validation is utilized to evaluate the performance of the proposed method. The whole PPI dataset is randomly divided into five subsets of roughly equal size, and each subset consists of nearly equal number of interacting and noninteracting protein pairs. Four out of these five subsets are used for training and the remaining one for test. This process is repeated five times such that each subset is used once and only once for test. The results are then averaged over the five runs to ensure the highest level of fairness.

The prediction performance of SVM predictor with matrix-based protein sequence representation across five runs is shown in Table 2. It can be observed from Table 2 that high prediction accuracy 90.06% is obtained for the proposed model. To better investigate the prediction ability of our model, we also calculated the values of sensitivity, precision, MCC, and AUC. From Table 2, we can see that our model gives good prediction performance with an average sensitivity value of 85.74%, precision value of 93.84%, MCC value of 82.03%, and AUC value of 95.28%. Further, it can also be seen from Table 2 that the standard deviation of sensitivity, precision, accuracy, MCC, and AUC is as low as 0.0094, 0.0098, 0.0064, 1.03, and 0.0064, respectively.

We further compared our method with those of Guo et al. [22], Zhou et al. [30], and Yang et al. [31], where the SVM, SVM, and KNN were performed with the conventional autocovariance, local descriptor, and local descriptor representation as the input feature vectors, respectively. From Table 2, we can see that the performance of all of these methods with different machine learning models and sequence-based feature representation methods are lower than ours, which indicates the advantages of our method. To sum up, we can readily conclude that the proposed approach generally outperforms the previous model with higher discrimination power for predicting PPIs based on the information of protein sequences. Therefore, we can see clearly that our model is a much more appropriate method for predicting new protein interactions compared with the other methods. Consequently, it makes us more convinced that the proposed method can be very helpful in assisting the biologist to contribute to the design and validation of experimental studies and in the prediction of interaction partners.

### 3.3. Comparison between the Proposed Model and AADC Method

The amino acid dipeptide composition (AADC) is a representation method for protein sequences that count the frequency of occurrence of adjacent pairs of amino acids. Similar to the proposed matrix-based protein sequence representation method, AADC only needs the information of protein amino acids; no attention is paid to the physicochemical properties of amino acids or other pieces of biological information about proteins. To demonstrate the performance of the proposed model, we further compared the proposed protein feature representation methods with AADC method.

The prediction performance of SVM predictor with the aforementioned two protein sequence representation across five runs is shown in Table 3. It can be observed from Table 3 that high prediction accuracy of 90.06% is achieved for the proposed model with Gaussian kernel function. To better investigate the prediction ability of our model, we also calculated the values of sensitivity, specificity, PPV, NPV, *F*-score, MCC, and AUC. From Table 3, we can see that our model gives good prediction performance with an average sensitivity value of 85.74%, specificity value of 94.37%, PPV value of 93.84%, NPV value of 86.89%, *F*-score value of 89.61%, MCC value of 82.03%, and AUC value of 95.28%. Further, it can also be seen from Table 3 that the standard deviation of accuracy, sensitivity, specificity, PPV, NPV, *F*-score, MCC, and AUC is as low as 0.0064, 0.0094, 0.0095, 0.0098, 0.0048, 0.0076, 0.0103, and 0.0064, respectively. The performance of the proposed model with other kernel functions including sigmoid function, polynomial function, and linear function is also demonstrated in Table 3.

In addition, the prediction performance of AADC based model is shown in Table 3. The AUC of the AADC model with Gaussian kernel is 0.9292, which is lower than that of the proposed model. The overall accuracy, sensitivity, specificity, PPV, NPV, *F*1 score, and MCC of AADC model are, respectively, 86.54%, 83.49%, 89.59%, 88.92%, 84.43%, 86.12%, and 76.66% as illustrated in Table 3. Hence, it can be seen that almost all evaluation measures of the proposed model are better than those of AADC method.

**Figure 2:**
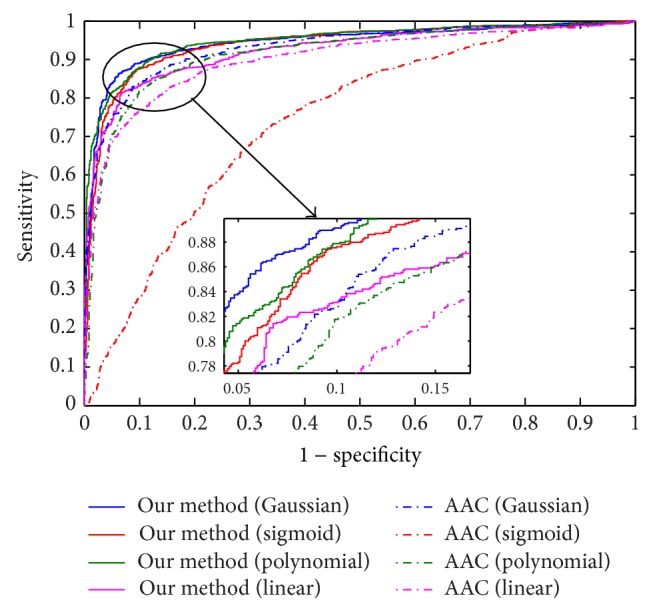
The ROC (receiver operator characteristic) curve illustrating the performance of different activation functions. The curve presents the true positive rate (sensitivity) against the false positive rate (1 − specificity).

We also conduct experiment to characterize the sensitivity (i.e., the size of true positives that can be detected by our method) and specificity (i.e. 1 − false positive rate) of the proposed approach for different activation functions (see Figure 2). The results in Figure 2 are reported using receiver operator characteristic (ROC) curves, which plot the achievable sensitivity at a given specificity (1 − false positive rate). Good performance is reflected in curves with a stronger bend towards the upper-left corner of the ROC graph (i.e., high sensitivity is achieved with a low false positive rate). We found that proposed method achieved over 89 percent detection rate with less than 10 percent false positive rate. The results demonstrate that the proposed matrix-based model can successfully classify positive and negative samples in all five activation functions that we investigated. Our algorithm can perfectly classify interacting and noninteracting protein pairs with only a few exceptions.

To sum up, considering the high efficiency as well as the good performance we can readily conclude that the proposed approach generally outperforms the AADC model with higher discrimination power for predicting PPIs based on the information of protein sequences. Therefore, we can see clearly that our model is a much more appropriate method for predicting new protein interactions compared with the other methods.

### 3.4. Comparing the Prediction Performance between Our Method and Other Existing Methods

In order to highlight the advantage of our model, it was also tested by* Helicobacter pylori* dataset. This dataset gives a comparison of proposed method with several previous works including phylogenetic bootstrap [32], signature products [24], HKNN [33], and boosting [34]. The methods of phylogenetic bootstrap, signature products, and HKNN are based on individual classifier system to infer PPIs, while the methods of boosting belong to ensemble-based classifiers.

The average prediction results of 10-fold cross-validation over five different approaches are demonstrated in Table 4. From Table 4, we can see that the average prediction performance, that is, sensitivity, precision, accuracy, and MCC achieved by proposed predictor, are 85.27%, 83.33%, 85.91%, and 75.53%, respectively. It clearly shows that our method outperforms all other individual classifier-based methods and the ensemble classifier systems (i.e., boosting). All these results demonstrate that the proposed method not only achieves accurate performance, but also substantially improves precision in the prediction of PPIs.

## 4. Conclusions

In this paper, we proposed an efficient and accurate learning technique, which utilizes the information of protein amino acid sequence order and distribution, for accurate identification PPIs at considerably high speed. It is well known that the order and distributions of dipeptide possess more pieces of information than those of amino acid dipeptide composition (AADC), so the main advantage is that this algorithm can extract more pieces of information hidden in protein primary sequences than AADC can. Then, the application of SVM predictor ensures reliable recognition with minimum error. Experimental results demonstrated that the proposed method performed significantly well in distinguishing interacting and noninteracting protein pairs. It was observed that the proposed method achieved the mean classification accuracy of 90.06% using fivefold cross-validation. Meanwhile, comparative study was conducted on the proposed method and other existing methods. The experimental results showed that our method outperformed these works in terms of classification accuracy.

## Figures and Tables

**Table 1:** The matrix-based representation for a protein amino acid sequence.

	*S*_1_ *S*_2_	*S*_2_ *S*_3_	*S*_3_ *S*_4_	*S*_4_ *S*_5_	⋯	*S*_*L*−1_ *S*_*L*_
AA	*a*_11_	*a*_12_	*a*_13_	*a*_14_	⋯	*a*_1,*L*−1_
AR	*a*_21_	*a*_22_	*a*_23_	*a*_24_	⋯	*a*_2,*L*−1_
AN	*a*_31_	*a*_32_	*a*_33_	*a*_34_	⋯	*a*_3,*L*−1_
⋮	⋮	⋮	⋮	⋮	⋮	⋮
VV	*a*_400,1_	*a*_400,2_	*a*_400,3_	*a*_400,4_	⋯	*a*_400,*L*−1_

**Table 2:** Comparing the prediction performance by the proposed method and some state-of-the-art works on the *yeast* dataset. Here, N/A means not available.

Model	Test set	SN (%)	PPV (%)	ACC (%)	MCC (%)
Proposed method	SVM	**85.74** ± **0.94**	**93.84** ± **0.98**	**90.06** ± **0.64**	**82.03** ± **1.03**

Guos' work	ACC	89.93 ± 3.68	88.87 ± 6.16	89.33 ± 2.67	N/A
	AC	87.30 ± 4.68	87.82 ± 4.33	87.36 ± 1.38	N/A

Zhous' work	SVM + LD	87.37 ± 0.22	89.50 ± 0.60	88.56 ± 0.33	77.15 ± 0.68

Yangs' work	Cod1	75.81 ± 1.20	74.75 ± 1.23	75.08 ± 1.13	N/A
	Cod2	76.77 ± 0.69	82.17 ± 1.35	80.04 ± 1.06	N/A
	Cod3	78.14 ± 0.90	81.86 ± 0.99	80.41 ± 0.47	N/A
	Cod4	81.03 ± 1.74	90.24 ± 1.34	86.15 ± 1.17	N/A

**Table 3:** Comparing the prediction performance by the proposed method and amino acid dipeptide composition method on the yeast dataset.

Methods	Kernel	Mean/std.	Testing							
			ACC	SN	SP	PPV	NPV	*F*1	MCC	AUC
The proposed method	Sigmoid	Mean	0.8734	0.8379	0.9092	0.9032	0.8474	0.8693	0.7784	0.9385
		Variance	0.0073	0.0093	0.0078	0.0087	0.0063	0.0088	0.0111	0.0071
	Gaussian	Mean	**0.9006**	**0.8574**	**0.9437**	**0.9384**	**0.8689**	**0.8961**	**0.8203**	**0.9528**
		Variance	0.0064	0.0094	0.0095	0.0098	0.0048	0.0076	0.0103	0.0064
	Polynomial	Mean	0.8963	0.8517	0.9408	0.9351	0.8639	0.8915	0.8134	0.9506
		Variance	0.0079	0.0072	0.0112	0.0118	0.0050	0.0085	0.0124	0.0061
	Linear	Mean	0.8642	0.8267	0.9016	0.8938	0.8389	0.8589	0.7646	0.9238
		Variance	0.0048	0.0098	0.0114	0.0103	0.0073	0.0052	0.0068	0.0038

AADC method	Sigmoid	Mean	0.6776	0.6726	0.6825	0.6792	0.6760	0.6758	0.5630	0.7343
		Variance	0.0088	0.0194	0.0098	0.0107	0.0136	0.0133	0.0062	0.0129
	Gaussian	Mean	0.8654	0.8349	0.8959	0.8892	0.8443	0.8612	0.7666	0.9292
		Variance	0.0065	0.0104	0.0047	0.0041	0.0119	0.0058	0.0095	0.0087
	Polynomial	Mean	0.8514	0.8196	0.8833	0.8754	0.8305	0.8465	0.7465	0.7540
		Variance	0.0063	0.0144	0.0078	0.0072	0.0110	0.0077	0.0090	0.3751
	Linear	Mean	0.8409	0.8150	0.8668	0.8597	0.8240	0.8367	0.7320	0.9021
		Variance	0.0060	0.0050	0.0146	0.0128	0.0070	0.0049	0.0080	0.0030

**Table 4:** Performance comparison of different methods on the *H. pylori* dataset. Here, N/A means not available.

Methods	SN (%)	PE (%)	ACC (%)	MCC (%)
Phylogenetic bootstrap	69.8	80.2	75.8	N/A
HKNN	86	84	84	N/A
Signature products	79.9	85.7	83.4	N/A
Boosting	80.37	81.69	79.52	70.64
Proposed method	**85.27**	**83.33**	**85.91**	**75.53**
